# *Methanosarcina* Play an Important Role in Anaerobic Co-Digestion of the Seaweed *Ulva lactuca*: Taxonomy and Predicted Metabolism of Functional Microbial Communities

**DOI:** 10.1371/journal.pone.0142603

**Published:** 2015-11-10

**Authors:** Jamie A. FitzGerald, Eoin Allen, David M. Wall, Stephen A. Jackson, Jerry D. Murphy, Alan D. W. Dobson

**Affiliations:** 1 Environmental Research Institute, University College Cork, Lee Road, Cork, Ireland; 2 School of Microbiology, University College Cork, Cork, Ireland; 3 School of Engineering, University College Cork, Cork, Ireland; 4 Science Foundation Ireland, Marine Renewable Energy Ireland (MaREI) Centre, University College Cork, Cork, Ireland; Fudan University, CHINA

## Abstract

Macro-algae represent an ideal resource of third generation biofuels, but their use necessitates a refinement of commonly used anaerobic digestion processes. In a previous study, contrasting mixes of dairy slurry and the macro-alga *Ulva lactuca* were anaerobically digested in mesophilic continuously stirred tank reactors for 40 weeks. Higher proportions of *U*. *lactuca* in the feedstock led to inhibited digestion and rapid accumulation of volatile fatty acids, requiring a reduced organic loading rate. In this study, 16S pyrosequencing was employed to characterise the microbial communities of both the weakest (R1) and strongest (R6) performing reactors from the previous work as they developed over a 39 and 27-week period respectively. Comparing the reactor communities revealed clear differences in taxonomy, predicted metabolic orientation and mechanisms of inhibition, while constrained canonical analysis (CCA) showed ammonia and biogas yield to be the strongest factors differentiating the two reactor communities. Significant biomarker taxa and predicted metabolic activities were identified for viable and failing anaerobic digestion of *U*. *lactuca*. Acetoclastic methanogens were inhibited early in R1 operation, followed by a gradual decline of hydrogenotrophic methanogens. Near-total loss of methanogens led to an accumulation of acetic acid that reduced performance of R1, while a slow decline in biogas yield in R6 could be attributed to inhibition of acetogenic rather than methanogenic activity. The improved performance of R6 is likely to have been as a result of the large *Methanosarcina* population, which enabled rapid removal of acetic acid, providing favourable conditions for substrate degradation.

## Introduction

While primarily a waste-treatment strategy, Anaerobic Digestion (AD) is increasingly being implemented as a viable renewable-energy technology, capable of converting diverse organic substrates into biofuels. In this respect, there is renewed interest in the use of seaweeds (macro-algae) as a substrate for biofuel production [1,2], though some technical problems associated with their use still need to be resolved [3].

In contrast to plants, seaweeds possess lower quantities of recalcitrant structural polymers (e.g. lignin, cellulose, hemi-cellulose), contain large reserves of accessible carbohydrates, and produce biomass via a rapid life cycle. However, they also possess unique compounds. *U*. *lactuca* can yield high levels of protein, sulphur and nitrogen; seaweeds typically also contain excess marine salts [4–8]. To improve biogas yields, pre-treatments, co-digestion, and alternative reactor configurations have been investigated for seaweeds [3]. Efficient management of AD via process parameters can also improve biogas yields, as well as helping to avoid toxic shock (e.g. rapid changes in pH, ammonia etc.), accumulation of intermediates (e.g. volatile fatty acids), or over/under-feeding of the reactor (i.e. maintaining an appropriate organic loading rate). However, these parameters provide only indirect information on biological processes within the reactor, and often must be re-evaluated at each new application, restricting informative comparisons and potentially obscuring underlying processes.

Recent reports have highlighted the need for microbial indicators of optimal AD performance as a prerequisite to allow “microbial-based management” of the process [9,10]. Thorough characterisation and a greater understanding of microbial populations and processes “driving” AD can better inform the design and operation of biogas reactors treating macro-algae and other novel feedstocks. Identifying these 'indicators' has been greatly aided by the use of molecular sequencing technologies, allowing metagenomic-based analyses of microbial community structures in various AD systems. These approaches have successfully been employed to monitor the development of AD communities over time [11,12] determine core motifs in AD community structure [13], and determine dominant methanogenic pathways which can be correlated to biogas yield [14]. Previous metagenomic studies on the use of algae as a biogas substrate have identified increases in the archaeal methanogenic order *Methanosarcinales* under addition of the macro-alga *Saccharina latissima* [15], the importance of *Methanosarcinales* in supporting diverse metabolic pathways in AD of the micro-alga *Scenedesmus obliquus* [16], and the importance of retaining methanogenic *Archaea* in AD of the macro-alga *Laminaria hyperborea* [17].

In a previous study, Allen and co-workers approached difficulties in digesting the macro-alga *Ulva lactuca* (sea-lettuce) through co-digestion with the proven and abundant substrate, dairy slurry. Six *U*. *lactuca-*slurry feedstock ratios were trialled over a nine-month period, with five of the reactors (R1 through R5) encountering total or partial inhibition through overloading of volatile fatty acids (VFAs), which was dependant on the quantity of *U*. *lactuca* supplied [18]. A sixth reactor (R6) saw no immediate inhibition, but instead demonstrated a slow decline in biogas yield, which could not be explained through process variables [18]. Here, we present a microbial analysis of these trials, investigating how AD of *U*. *lactuca* shaped archaeal and bacterial populations in the best (R6) and worst (R1) performing reactors, with a particular focus on methanogenic processes. A taxonomic time-series was constructed which illustrates how microbial community structure and activity diverged between R1 and R6, suggesting two explanations for loss of methanogenic activity and a mechanism for *Methanosarcina* improving reactor stability. Constrained canonical analysis (CCA) revealed the most significant effects of *U*. *lactuca* on microbial community structure and on predicted metabolic activity. To our knowledge, this is the first application of 'next-generation' 16S community sequencing to monitor microbial community structures involved in anaerobic digestion of green seaweeds (*Chlorophyta*).

## Materials and Methods

### Biogas reactor configuration

**Fig 1:**
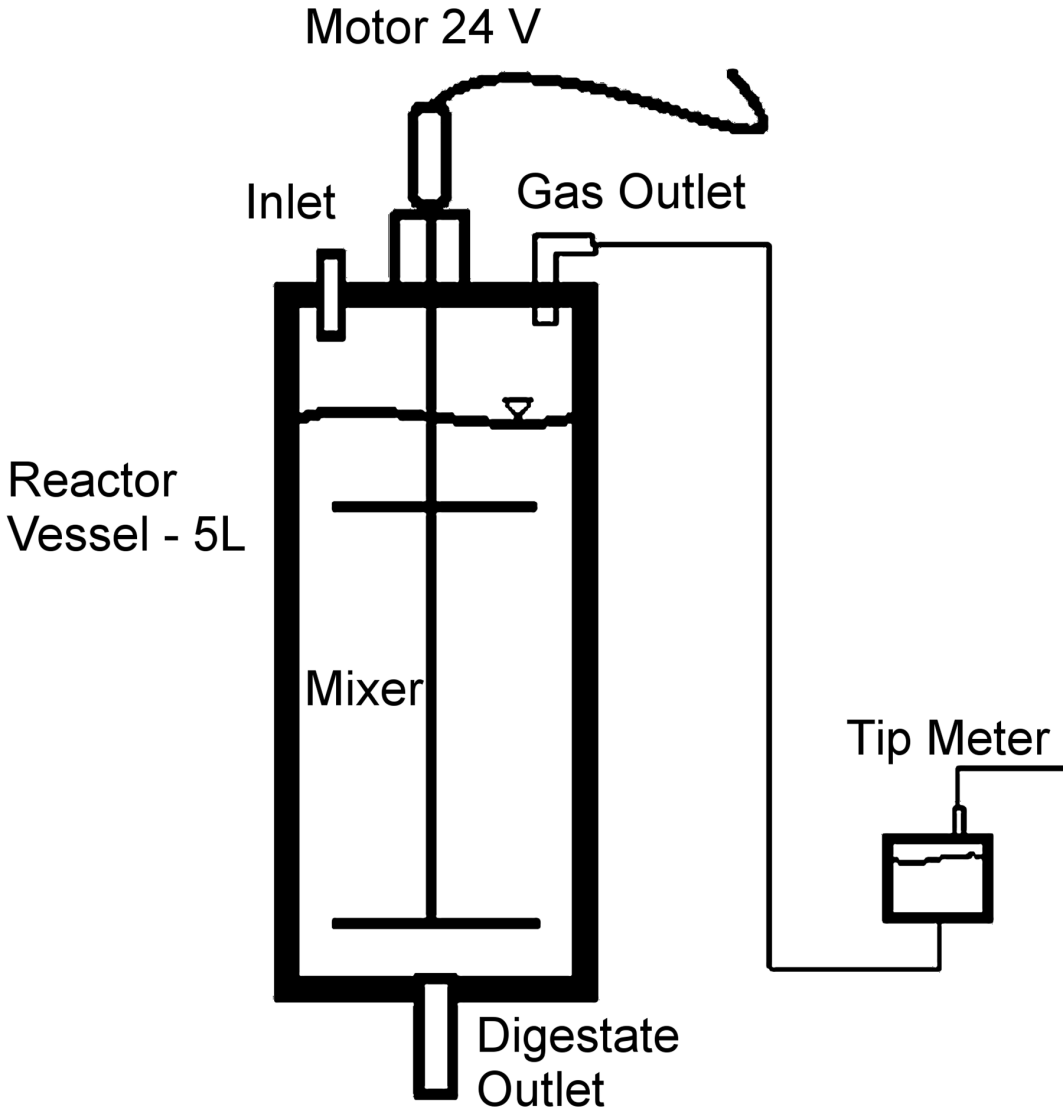
Schematic of Reactor set-up for R1 and R6.

A total of six, 5L one-step continuously stirred-tank reactors (CSTRs) were operated in parallel digesting mixes of *Ulva lactuca* and dairy slurry for a period up to 42 weeks at a constant temperature of 37°C. Three reactors treated dried *U*. *lactuca* in co-digestion mixes of 25, 50 and 75% with dairy slurry. A further 3 reactors co-digested fresh *U*. *lactuca* with slurry in the same ratios. Regular feeding and removal of substrate allowed a constant 4 L working volume, with an initial organic loading rate (OLR) of 2 kg VS m^3^ d^-1^. Of the 6 reactors, 3 failed to obtain steady state biogas production, 2 achieved steady state production profiles but incurred high levels of VFA-based inhibition, while the final reactor achieved satisfactory yields. Inhibition was characterised by variable levels of VFA and biogas yield, and an inability to maintain high rates of substrate input. Reactors were operated in the configuration represented in Fig 1. Previous work [4] assessing the optimal bio-methane potentials (BMP) for *U*. *lactuca*/slurry feedstocks allowed evaluation of reactor output.

Reactor 1 (R1: digesting 75% dried *U*. *lactuca*, 25% dairy slurry) provided the longest running example of *U*. *lactuca*-inhibited digestion, while Reactor 6 (R6: digesting 25% fresh *U*. *lactuca*, 75% dairy slurry) was the best performing reactor, with stable VFA concentrations and favourable yields at an OLR of 2.5 kg VS m^3^ d^-1^. R1 and R6 were subsequently chosen as best and worst case examples of *U*. *lactuca* co-digestion. Reactor R1 was operated for a total of 40 weeks. Initially an OLR of 2 kg VS m^3^ d^-1^ was used for R1, however failure to reach the designated yields after the first hydraulic retention time (HRT) and the increase in VFA concentration resulted in the OLR being reduced to 1 kg VS m^3^ d^-1^, with subsequent steady-state biogas production being achieved. R6 was also operated for 40 weeks. An OLR of 2 kg VS m^3^ d^-1^ was successfully maintained for R6 after a period of 3 HRTs, with OLR then being increased to 2.5 kg VS m^3^ d^-1^. Steady state biogas production was achieved throughout this period. A gradual decline was observed in the final HRT for R6 without a corresponding increase in VFA or ammonia concentrations accounting for this reduction [18]. The decision to increase OLR was determined by two factors: the relationship between VFA concentrations and reactor performance, and the biomethane conversion efficiency (B_eff_). The effect of VFAs was determined using the Nordmann method [19] commonly known as the FOS:TAC ratio, measuring volatile organic acids and total inorganic carbonate. Operational ranges set out by this method dictate whether the reactor is being over, under or fed satisfactorily. The biomethane conversion efficiency (B_eff_) is the specific methane yield (SMY) of that reactor in continuous digestion divided by the biochemical methane potential (BMP) yield obtained from a 30 day batch test on that exact substrate. Values closer to or higher than 1 are desirable, reflecting optimum conversion of feedstock to biogas. A comprehensive detailing of the laboratory methods used to analyse all the environmental parameters within R1 and R6 has been previously described [18].

### Sampling and Molecular Methods

Reactor sludges were sampled on a weekly basis, and frozen at -80°C until further analysis. For R1, weeks 1, 5, 13, 20, 30 and 39 were selected as representative time-points, spanning five retention times. For R6, weeks 1, 5, 13, 21 and 27 were selected as time-points, spanning four retention times. Sludge from these 11 time-points was processed with the PowerSoil DNA extraction kit (MoBio, CA, USA) with the following protocol modifications: 1) initial 'wet-spin' (30 seconds at 10,000 g) to remove an excess liquid fraction prior to cell lysis; 2) 3x cycles of 10 minute bead-beating followed by 5 minutes chilling at -20°C; 3) 2x washes of elution buffer. For each time-point, triplicate sludge-samples were taken from each reactor. From each of these, three separate DNA extractions were performed, and then combined in equimolar quantities to ensure representative sampling. Extractions were quantified spectrophotometerically (ND-1000, Thermo-Fisher, DE, USA) and viewed on 1% agarose gel with ethidium bromide (1μg/ml).

16S gene sequences were amplified from the DNA extracts using 11 pyrosequencing PCR primers with the following motifs: adapter sequence (Roche-454 Lib-A and Lib-B chemistry); key sequence (TCAG); Roche-454 pyrosequencing MIDs 1–10 and 12 inclusive; and 16S universal primers U-789F (5' TAGATACCCSSGTAGTCC 3') and U-1053R (5' CTGACGRCRGCCATGC 3') [20]. A program of initial denaturation at 94°C for 5 minutes, followed by 26 cycles of 30 seconds denaturing at 95°C, 30 seconds annealing at 53°C, and 45 seconds of extension at 72°C, with a final extension step of 72°C held for 6 minutes was followed. Products in the expected size range were extracted using a gel extraction kit (QIAGEN, Manchester, UK), which required subsequent use of a PCR purification kit (QIAGEN, Manchester, UK). Each DNA extract was amplified in triplicate, then pooled in equimolar quantities to produce 11 community samples, which were then pyrosequenced by MACROGEN (Seoul, Republic of Korea).

### Bioinformatic Analysis

Denoising was performed in Acacia [21] before import into the Quantitative Insights Into Microbial Ecology (QIIME) software pipeline [22] for de-mulitplexing, chimera removal, aligning, taxonomic assignment and exploratory analyses. Sequences were split into sample libraries; Chimera filtering was carried out using USEARCH v6.1 [23]; Alignments and taxonomic assignments were carried out with reference to the Silva 111 Database release [24] at 97% similarity using PyNast [25] and the RDP Classifier 2.2 [26]; Tree building was carried out using FastTree [27]. Beta diversity was calculated using UniFrac [28] and 3D PCoA plots generated by Emperor [29].

Sequence data was combined with reactor process data from [18] within the *R* statistics program [30]. *R* packages *vegan* [31] and *phyloseq* [32] were used to subset population abundances by sample and/or reactor environment, and to perform statistical analysis.

Greengenes release 13.5 [33] was used to perform closed-reference OTU picking in QIIME prior to generating metabolic predictions from the Kyoto Encyclopedia of Genes and Genomes (KEGG; release 73.1 [34]) with the HMP Unified Metabolic Analysis Network (HUMAnN) [35] package. Significant differences between the two reactors were calculated using the LDA Effect Size (LEfSe) resource [36] on the Huttenhower Galaxy resource [37–39] to analyse taxonomic and predicted metabolic data. To reduce spurious inferences on metabolic activity, a more conservative LDA threshold of 3 was used.

Sequence data was deposited in the MG-RAST database under project number 14106, and is publicly available at the URL http://metagenomics.anl.gov/linkin.cgi?project=14106.

It should be noted that although primers used in this study [20] continue to see use in similar investigations [40–42], primers are continuously refined to increase coverage as observed microbial diversity expands. Similarly, methodologies that minimise bias [43], and reference databases with improved taxonomic and metabolic representation continue to be developed (e.g. Silva, KEGG). As such, the characterisation of communities in this study is necessarily incomplete and likely to contain errors at lower limits of taxonomic resolution–metabolic characterisation in particular is still in its infancy, with prediction best employed as an exploratory or complementary analysis. Improved, robust characterisations of AD community members are anticipated from future studies, employing updated biological data and methodologies.

## Results and Discussion

A previous study trialled continuous anaerobic digestion of varying ratios of *Ulva lactuca* and dairy slurry, demonstrated severely inhibited biogas production at higher *U*. *lactuca* loading levels [18]. To determine potential causes of this inhibition, the microbial community profiles of two reactors digesting contrasting ratios of *U*. *lactuca* and dairy slurry were characterised and compared, with the overall aim of identifying significant 'biomarker' species or metabolic activities which differentiated successful and inhibited digestion of *U*. *lactuca*. Detailed accounts of reactor setup and performance have been provided by [18].

### Process results of biogas reactors, R1 and R6

Previous characterisations [4] of feedstocks predicted ideal biomethane yields of 210 and 183 L per kilogram of volatile solids (kgVS^-1^) for R1 and R6 respectively. R1 started at an OLR of 2 kgVSm^-3^d^-1^, changing to 1 kgVSm^-3^d^-1^ at Week 6 and 1.5 kgVSm^-3^d^-1^ at Week 33 in response to high VFA levels. R6 started at an OLR of 2 kgVSm^-3^d^-1^, elevating to 2.5 kgVSm^-3^d ^-1^ at Week 22. A comparative summary of the reactors is provided in Table 1.

**Table 1:** Highlights of results of semi continuous digestion trials.

**Setup**	**CSTR R1**			**CSTR R6**	
% *U*. *lactuca*	75 (dried)			25 (dried)	
TS (%)	29.61			10.55	
VS (%)	18.42			7.22	
BMP (CH_4_ kg VS^-1^)	210 ± 6.3			183 ± 7.8	
Temperature (°C)	37			37	
**Parameters**	**HRT 1**	**HRT 2**	**HRT3**	**HRT 1**	**HRT 2**
OLR (kg VS m^3^ d^-1^)	2	1	1.5	2	2.5
Methane content (%)	33	47	47	51	52
SMY (CH_4_ kg VS^-1^)	83.31	176.77	145.21	178.11	170.46
B_eff_	0.4	0.84	0.69	0.95	0.93
VFA (mg l^-1^)	4954	4135	4355	1955	1720
FOS:TAC	0.56	0.34	0.43	0.39	0.3
TAN (mg l^-1^)	3443	5250	5300	2168	3000

Abbreviations: B_eff_: Biomethane conversion efficiency; BMP: Biomethane Potential; CSTR: Continuously-Stirred Tank Reactor; FOS:TAC: Buffering capability of solution; HRT: Hydraulic Retention Time; OLR: Organic Loading Rate; SMY: Specific Methane Yield; TS: Total Solids; VFA: Volatile Fatty Acids; VS: Volatile Solids

At steady-state operation, the specific methane yield (SMY) per kgVS^-1^ was similar between the two reactors: R1 and R6 on average produced 177 and 174 L CH_4_ L kgVS^-1^, respectively [18]. Despite these similar volumes, the R1 feedstock had a higher potential biomethane output (as above; R1: 210 L versus R6: 183 L kgVS^-1^ [4]): R1 therefore exhibited lower efficiencies (B_eff_ = 0.4, 0.69, 0.84) compared to R6 (B_eff_ = 0.95, 0.93). However, the biggest difference between reactor performances was rate of substrate conversion.^,^ At OLRs 1 and 1.5 kgVSm^-3^d^-1^, R1 produced biomethane at efficiencies of 0.84 and 0.69; at OLRs of 2 and 2.5 kgVSm^-3^d^-1^, R6 was converting more substrate and at consistently higher efficiencies of 0.93–0.95.

### Process Inhibitors

#### Volatile Fatty Acids

VFA accumulation can occur as a product of instability [44], can be transitional [45–47] and can even have little to no effect on biogas production [48]. Initial accumulation of iso-valeric and acetic acids was seen in both reactors: the relative difference between build-ups (initially three-fold higher in R1; higher thereafter) suggests this was due to hydrolysis and fermentation of the most accessible fractions of *U*. *lactuca*.

#### NH3

The recommended ratio of carbon to nitrogen (C:N ratio) for anaerobic digestion is between 20:1 and 30:1. C:N ratios for *U*. *lactuca* range between 7:1 [18] and 14.5:1 [5]. C:N ratios for feedstocks in this study were 10.2:1 for R1 and 17.1:1 for R6, with higher values reflecting addition of slurry (C:N ratio often >20:1 [49]). Proteins contribute nearly all of the nitrogen in *U*. *lactuca* [8], entering solution as free ammonia (NH_3_) or the ammonium ion (NH_4_^+^). Elevated pH, temperature, and headspace partial pressure increase concentration of the uncharged NH_3_ state. At sufficiently high concentrations NH_3_ diffusion across cell membranes can inhibit the biogas process by causing loss of cellular potassium, de-potentiating the cell membrane, and accumulating in the cytoplasm [50]. Ammonia inhibition is well documented in methanogens [50–53], affecting other taxa to a greater or lesser extent. Pure cultures of methanogens remain viable at TAN levels up to 10,000 mg/L but have been documented to decline at a range of TAN levels between 1,700 to 6,000 mg/L when a part of a reactor community. Differential responses between hydrogenotrophic and acetoclastic methanogens are documented but contradictory (see reviews [54] and [55]).

#### Mineral salts

An inhibitory role for salts has long been recognised in anaerobic digestion [56]. Cations (e.g. Na^+^, Ca^2+^, Mg^2+^, K^+^) affect biogas production in a charge-dependent manner, possibly by inhibiting a Na^+^ export channel necessary for the final methanogenic reaction [57]. However, complex and proportionate mixes of cations can offset the inhibitory effects of one another [56,58], as well as ameliorating inhibition of the biogas process due to ammonia [53] and VFA inhibition [59]. Pre-trial characterisations showed slurry to have low (< 2,000 mg/L) total mineral content, while fresh *U*. *lactuca* provided 5,220, 5,310 and 9,950 mg/L of Mg^2+^, Na^+^ and Ca^2+^ respectively. Monitored levels of Cl^-^ infer that salt-loading was significantly higher in R1, with a two-fold difference between R1 and R6 at close of trial (~10,300 and ~5,400 mg/L respectively). Reported inhibitory levels of Na^+^ and Ca^2+^ vary, with lower estimates of inhibition registering from 5,000 mg/L upwards [54]. Community acclimatisation and/or later inhibitory onset are likely, due to gradual accumulation and the variety of salts.

### Community Composition

#### Sequencing results and diversity measures

Pyrosequencing generated 270,111 raw sequences, which following denoising in Acacia and processing in QIIME resulted in 89,251 sequence reads (average length: 244bp) being produced, with an average of 8,114 reads per trial time-point. To ensure representative samples from both reactors, diversity metrics were calculated to estimate sensitivity to species diversity (Chao1 index) and species abundances (Simpson's Index). Rarefaction curves of these indices indicate that the most abundant species were thoroughly characterised in this study (see S1 Fig). However, rarefaction curves also indicate that a large number of low-abundance *Archaea*, *Bacteria* and unidentified taxa remain undetected due to insufficient depth of sequencing. Finally, both diversity indices (Chao1, Simpson's) decreased in later samples, suggesting the maturation of trophic systems in both reactors, where 'surplus' diversity is marginalised beyond the sequencing threshold.

### Community Makeup

The QIIME pipeline identified 2,824 Operational Taxonomic Units (OTUs) in the 89,251 sequence reads. Singleton and doubleton OTUs (abundances < 3 reads) were discarded to reduce statistical noise, leaving 1,320 OTUs (82,914 sequence reads). Of the 1,320 OTUs, 1,057 were present in R1 and 955 in R6. Taxonomic alignments provided by Silva (release 111) identified 2 phyla, at least 4 classes, 5 orders, 7 families and 8 genera of *Archaea* (20 OTUs, 9,010 sequences), and at least 34 phyla/candidate phyla, 44 classes, 86 orders, 124 families and 190 unique genera of *Bacteria* (1,206 OTUs, 73,185 sequence reads). Lower taxonomic classifications could not be assigned to 16% of *Bacteria* families and 53% of *Bacteria* genera. A final 94 OTUs remained unidentified and were not assigned t*o Bacteri*a o*r Archaea*. Unassigned taxa comprised 1% of sequence reads (72 OTUs) from R1, and <1% of reads (42 OTUs) from R6. A complete description of community abundances is provided as supplementary data in S1 Table.

#### Archaeal communities

*Methanosarcina* was the most abundant genus in this study (7 OTUs, 9.7% of all sequence reads), the majority of which originated from R6 (9.5% of all sequence reads). Large *Methanosarcina* populations are known to effectively buffer against fluctuations in substrate availability, preventing accumulation or shock loading of acetic acid [60,61]. *Methanosarcina* has a documented tolerance for acetic acid up to 15,000 mg/L, and a higher tolerance for changes in pH and salt (see review in [62]) than hydrogenotrophic counterparts. *Methanothrix*, an obligate acetoclast [63], was scarce or absent in this study, likely out-competed by the higher growth rate of *Methanosarcina* at non-limiting acetate concentrations [47,60,64], or inhibited by salt [54] or ammonia [52–55].

Hydrogenotrophic methanogens (*Methanoculleus*, *Methanobrevibacter*, *Methanobacterium*, *Methanocorpusculum*, *Methanospirillum* and *Methanosphaera* in this study) are commonly found in anoxic sediments [65], as gut flora [66–68], and in AD reactors where they sometimes dominate [13,69]. However, most archaeal OTUs were observed at consistently low frequencies (<0.5% of total sequence reads respectively), often disappearing below the threshold of sequencing coverage.

#### Bacterial communities

Bacterial components of these reactors are typical of biogas communities, while some key and accessory species are associated with marine or salt environments. The most abundant phylum was *Firmicutes* (565 OTUs, 36% of all sequence reads), containing many groups known to hydrolyse polymers (e.g. cellulose, lignin, polysaccharides, proteins: *Lachnospiraceae*, *Peptostreptococcaceae*, *Ruminococcaceae*), ferment carbohydrates (e.g. saccharides, amino acids, organic molecules: OPB54, *Gelria*, *Christensenellaceae*), and produce organic acids as metabolic endpoints (i.e.: acidogens: *Sedimentibacter Tissierella*, *Syntrophomondaceae*). *Firmicutes* are major components of anaerobic environments such as digesters [13,69,70] and alimentary tracts [71,72], and in this study accounted for over a third of sequences in both reactors: in short, they are highly diverse, widely distributed, and understood as essential components of anaerobic digestion.

The second-most abundant phylum, *Bacteroidetes* (126 OTUs, 16% of all sequence reads), is also frequently detected in anaerobic reactors, with important roles as fermenters and acidogens. In particular, species from the family *Porphyromonadaceae* (9% of all reads) are known to be involved in the degradation of proteins and amino acids, eschewing saccharides (genera *Petrimonas* [73] and *Proteiniphilum* [74]).

Phylum *Proteobacteria* (203 OTUs, 13% of sequence reads) comprises the most diverse known taxonomic group of the *Bacteria* to date. The sub-ordinate classes *Alpha*- and *Gamma-Proteobacteria* contributed 3% and 7% of reads in this study respectively, with remaining proteobacterial classes totaling 3%. *Proteobacteria* are typical residents of anaerobic digesters [13,69,75], known to incorporate nitrogen and/or sulphur as electron acceptors in the metabolism of varied carbohydrates (e.g.: *Nitrosimonas*, *Nitrobacter*). However, some species observed here are unexpected inclusions, with described preferences for aerobic metabolism (in some cases obligate: *Rhodobacteraceae*, *Granulosiococcaceae*, *Nannocystineae;*) and a high propensity for saline and marine environments (water: *Rhizobacteraceae*; sediments: *Desulfomicrobium*; seaweeds and plants: *Alteromonadaceae*, *Nannocystinaceae*, *Granulosiococcaceae*). As such, their presence in this study likely reflects persistent contributions from the *U*. *lactuca* feedstock alongside species typical of a biogas digester habitat.

Phylum *Spirochaetes* (47 OTUs and 6% of sequence reads in this study) are diverse, highly motile, frequently anaerobic bacteria, but metabolic information on this phylum in anaerobic digesters is somewhat limited despite being frequently encountered in low or medium abundances. They have been characterised both as acetogens [76,77] and acetoclasts assisting methanogenic activity (as Syntrophic Acetate-Oxidising Bacteria) [78].

Phylum *Synergistetes* comprised 6% of all sequence reads and 34 OTUs. *Synergistetes* are typically seen at lower abundances in a wide variety of environments [79], in syntrophic associations with hydrogenotrophic species (e.g. methanogens). A possible role in these reactors is likely to be oxidising amino acids as a substrate in the presence of methanogens [80,81].

Most phyla were present at much lower levels (< 2% of reads): Phylum *Chloroflexi* contains fermentative, acido- and acetogenic, obligate and facultative anaerobes seen in anaerobic digesters and hot springs respectively, and requires removal of hydrogen which suggests syntrophic roles [82]. Phylum *Tenericutes* is represented by *Acholeplasma* spp.- poorly characterised sugar fermenters [83]; Species from Phylum *Actinobacteria* contain many heterotrophic fermenters including lipidophiles, and obligate marine-associated species [84]; Phylum *Acidobacteria* species are uncharacterised but similar to sequences recovered from petrochemical-contaminated aquifers (isolate BPC102, NCBI accession AF154083.1); Taxa from Phylum *Armatimonadetes* are expected to be chemo-heterotrophs, and are suggested to associate with degradation of photosynthetic biomass [85].

Although the eleven phyla outlined above describe over 94% of all sequence reads, the remaining *Bacteria* (only 6% of reads, 135 OTUs) correspond to at least a further 26 phyla/candidate phyla, again reflecting the huge diversity in anaerobic reactor communities.

### Relating Community Makeup and Process Variables

**Fig 2:**
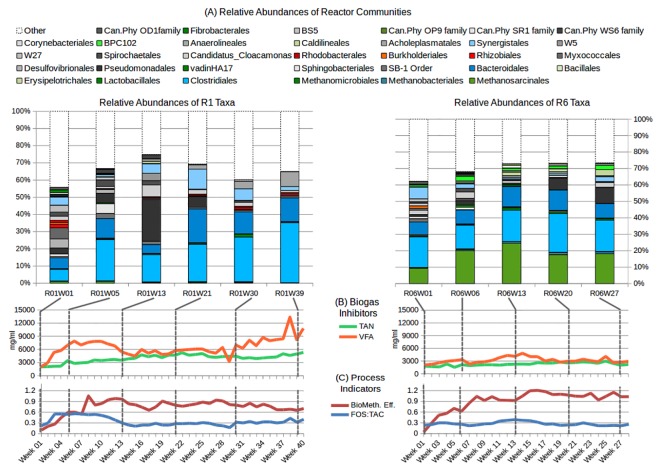
Interaction between community strudcture (at Order-level taxonomy) and major process variables. (A) Differences in reactor operation induce different community structures: R1, which struggled under heavy *U*. *lactuca* loading, developed larger fermenting populations and a lack of methanogens; R6, digesting less *U*. *lactuca*, retained large *Methanosarcina* populations even at higher OLRs. Referencing taxa abundances against levels of principal process inhibitors TAN and VFA (B), and indicators FOS:TAC and B_eff_ (C) illustrates the connection between community composition and biogas performance. Taxa which comprised less than 2% of sequence reads for all time-points are coalesced to 'Other' for convenience of viewing. Abbreviations: Total ammoniacal nitrogen (TAN), volatile organic acids (VOA), buffering ratio (FOS:TAC) and biomethane conversion efficiency (B_eff_). Taxa in red/orange represent biomarkers for R1; taxa in green represent biomarkers for R6; taxa in in blue contain biomarkers for both reactor setups (diverse Clostridiales and Bacetroidales).

A comparison of relative abundances for major Archaea and Bacteria (A), changes in levels of biogas inhibitors TAN and VFA (B), and biogas indicators B_eff_ and FOS:TAC (C) is given in Fig 2 for all time-points sampled in this study.

#### Changes in R1 community makeup

Week 1 conditions were initially favourable for R1 at an OLR of 2 kg VS m^3^ d^-1^, albeit with slightly elevated TAN and VFA levels (~2,000mg/L apiece). Community abundances were relatively balanced between hydrolysers, fermenters and acido-/acetogens (*Clostridiales*, *Bacteroidales*, *Desulfovibrionales*, *Synergistales*), with environmental inclusions (*Rhizobiales*, *Rhodobacterales*, *Myxococcales*) characteristic of slurry, *U*. *lactuca*, or marine sources.

Until Week 5, *Methanosarcina* abundances held at half (~1%) of all R1 archaeal sequence reads (~2%), suggesting conditions for acetoclasts were initially favourable. Canonical cellulose and protein degraders proliferated (*Ruminococcaceae*, *Lachnospiraceae*, *Proteiniphilum*). As TAN approached 3,500 mg/L, early accumulation of acetic and iso-valeric acid shifted to a sudden peak in iso-valeric acid (3,500mg/L) and depletion of acetic acid after Week 5. To reduce VFA content, OLR was reduced to 1 kg VS m^3^ d^-1^ in Week 7, while Cl^-^ levels passed 5,000mg/L.

Week 13 sequence reads showed a sharp rise in abundance of the *Pseudomonadales* genus *Psychrobacter* to 25%, alongside catabolism of accumulated iso-valeric acid to propionic and acetic acid. Associated with cold marine environments, *Psychrobacter* is likely to reduce amino and organic acids to acetic acid [86], suggesting an important role in continuous digestion of *U*. *lactuca* and slurry. However, *Methanosarcina* abundances were negligible (<0.1% of sequence reads) and not detected at end of trial, despite stable reactor conditions (FOS:TAC 0.21–0.31 until Week 26), a lack of inhibitory VFAs (<4,000 mg/L [62], and favourable levels of acetic acid for that genus (1100–1300 mg/L [62]; evidenced by similar concentrations in R6, Week 13). Hydrogenotrophic *Methanobrevibacter* and *Methanoculleus* were then the dominant Archaea in R1, at <1% of sequence reads.

Metabolism of accumulated propionic acid by Week 21 coincided with receding *Psychrobacter* abundance and expansion of hydrolysing and fermenting populations emphasising protein/amino acid metabolism and acetogenesis (OPB54, *Ruminococcaceae*, *Peptostreptococcaceae*, *Proteiniphilum*, *Aminobacterium*). TAN continued to increase (~4,700mg/L) alongside steady levels of acetic acid (~1,000 mg/L) as the main VFA. Hydrogenotrophic methanogens persisted at low levels (<1% sequence reads).

After peaking at Week 23 (~5,000mg/L), TAN stabilised by Week 30 (~4,000mg/L) while acetic and propionic acid had re-accumulated (~2,300 mg/L and ~500 mg/L respectively). Despite receding TAN, hydrogenotrophic methanogens declined further, with small shifts in bacterial populations from likely peptide (*Aminobacterium*, *Proteiniphilum*, *Psychrobacter*, *Peptostreptococcaceae*) to polysaccharide metabolisers (*Acholeplasmataceae*, *Ruminococcus*, OPB54).

Increasing OLR to 1.5 kg VS m^3^ d^-1^ at Week 34 exacerbated accumulation of TAN (+5,000mg/L), Cl^-^ (~6,800mg/L), and VFAs (chiefly acetic and propionic acid: ~3,200 and ~700mg/L respectively; FOS:TAC >0.4; declining biogas output). By Week 39, OPB54 (36% of sequence reads), *Proteiniphilum* (13%) and *Acholeplasmataceae* (9%) represented the most relatively abundant populations while Archaea contributed only 0.3% of sequence reads.

#### Changes in R6 community makeup

Initial levels of VFA and TAN in R6 were similar to R1, with accumulation of acetic and iso-valeric acid at lower levels, and large hydrolysing, fermenting and aceto-/acidogenic populations (*Clostridiales*: 32% of sequence reads, *Bacteroidales*: 10%, *Synergistales*: 8%). Notably, *Methanosarcina* was considerably more abundant at Week 1 (10% of sequence reads, as compared to 1% in R1). This may reflect a rapid acclimatisation to substrate (uncharacteristic of methanogens), or contribution from the three-fold higher slurry portion. R6 Archaea were also more diverse, including *Methanspirillum*, *Methanocorpusculum*, *Methanomasciliicoccus*.

Week 6 saw TAN rise above 2,000mg/L, with iso-valeric acid quickly metabolised to acetic acid. *Methanosarcina* relative abundance doubled to 22% of sequence reads, while *Clostridiales* and *Synergistales* taxa showed some decline in relative abundance.

Cl^-^ levels passed 5,000mg/L at Week 10. Week 13 represented the high point in biogas production, acetic acid availability, and *Methanosarcina* abundance (24% of sequence reads), alongside diverse bacterial populations with low, evenly-distributed abundances. The largest populations were acetogenic gut-associated saccharide fermenters (*Christensenellaceae*, *Rikenellaceae*: 4–6%), cellulose (*Ruminococcaceae*, *Lachnospiraceae*: 4–5%) and peptide (*Peptostreptococceae*, *Proteiniphilum*, *Sedimentibacter*: ~3%) degraders. Crucially,. Subsequent rises in propionic (700mg/L) and iso-caproic acids (600mg/l) were rapidly catabolised to acetic acid.

With TAN rising (~2,500mg/L) and a decrease in B_eff_ at Week 20, initially abundant bacterial taxa (*Peptostreptococcaceae*, *Lachnospiraceae*, *Christensenellaceae*, *Rikensellaceae*) were replaced by functionally similar populations (*Ruminococcaceae*, *Proteiniphilum*, *Psychrobacter*, OPB54) while *Methanosarcina* relative abundance decreased (18%) in conjunction with limiting acetic acid, similar to perturbation in the R1 community. An otherwise stable methanogen population (1.4%) suggests biogas obstruction prior to methanogenesis; sudden elevation of valeric acid (~500mg/L) implicates disrupted acetogenesis. Cl^-^ levels peaked at Week 21 (~6,800mg/L), but decreased thereafter (~6,000mg/L).

TAN peaked at 3,000mg/L in Week 25 before stabilising to ~2,000mg/L by Week 27, despite an increased loading rate of 2.5 kg VS m^3^ d^-1^. Abundances shifted towards larger, mono-typic populations of fermenters and acidogens, displacing degraders of cellulose and proteins, possibly in response to increased substrate availability. Relatively ideal reactor conditions (FOS:TAC 0.22–0.24; free ammonia and chloride below inhibitory levels; VFA concentrations below inhibitory levels despite an increased OLR [54,62]) and stable levels of *Methanosarcina*, combined with accumulated higher VFAs despite limiting acetic acid again suggest some inhibition of acetogenesis rather than methanogenesis is responsible for the decreasing yield seen in later R6 time-points.

### Statistical Resolution and Constrained Analysis

#### Taxonomic characteristics

To improve characterisation of the different microbe communities digesting slurry/*U*. *lactuca* mixes, the LDA (Linear Discriminant Analysis) Effect Size package (LEfSe, [36]) was used to detect taxa characteristic of digestion at high (R6) or low rates (R1), acting as potential 'biomarkers' for either setup. A complete LDA output for taxonomy is provided as supplementary data in S2 Table.

Taxa characteristic of R1 show a strong affinity for marine environments and/or halotolerance. Additionally, most were originally isolated from marine sources; three from *Ulva* species or other seaweeds (*Maritalea*, *Arenibacter*, *Alteromonadaceae*). Several are aerobes or facultative aerobes (*Nitratireductor*, *Altermonadaceae*) and many show degrees of fermentative and/or acidogenic activity. The most significantly associated taxa (LDA effect ≥4, α ≤0.05) are from the *Actinobacteria* (*Micrococcales*), *Alpha*-*Proteobacteria* (*Devosia*, *Nitratireductor*, *Rhizobium and Rhodobacteraceae sp*.), *Beta-Proteobacteria* (*Hydrogenophaga* and *Limnohabitans*), *Bacteroidetes* (*Proteiniphilum*) and *Firmicutes* (*Alkaliphilus*, *Bacillales*, *Lutispora*, *Syntrophomonadaceae*, *Tepidanaerobacterales*, *Tissierella*) phyla. As well as known fermenters, acidogens (*Proteiniphilum*, *Firmicutes*) and syntrophs (*Firmicutes*), these taxa suggest diverse saccharide use, and use of alternate electron acceptors (nitrogen, sulfur) detrimental to biogas production (*Alpha-* and *Betaproteobacteria*).

Indicators of the R6 environment were more closely linked to anaerobic digestion, but retained some associations with marine and saline habitats. The most significantly associated taxa (LDA effect ≥ 4, α ≤0.05) are more commonly anaerobic and documented as hydrolysers (*Alkaliflexus*, *Caldilineae*, *Lachnospiraceae*, *Proteiniphilum*, *Ruminococcaceae*), fermenters (*Caldilineae*, *Desulfomicrobia*) and acetogens (*Alkaliflexus*, *BPC102*, *Caldilinea*, *Christensenellaceae*, *Syntrophomonas*, *etc*.), as well as including three Archaea: the acetoclastic *Methanosarcina* and hydrogenotrophic *Methanobacterium* and *Methanobrevibacterium*. Most methanogens were not significant indicators, as abundances were similar between reactors.

#### Predicted metabolic characteristics

Attributing reactor performance to specific microbial populations is problematic, partially due to resource-intensive technologies necessary to profile metabolic activity, which may be unsuited to industrially scaled applications (e.g. mRNA/cDNA libraries, metabolic isotope analysis). A novel compromise afforded by metagenomics is to cross-reference taxonomic information (e.g. 16S sequence data) with a database of known metabolic capabilities, and compute inferred metabolic profiles which may help explain activities in a microbial community. Characterisation of functionality through inferred metabolism has been demonstrated in medical, ecological and biofuel contexts: identifying microbial metabolisms likely to improve dietary dysfunction [87]; demonstrating differential microbial activities in healthy and compromised habitats [88]; and predicting and confirming enriched cellulolytic activity in microbial lignocellulose degradation [89]. By highlighting the metabolic capabilities of an inoculum or sludge, the same approach applied to AD has the potential to provide a more informed characterisation of biogas conditions, helping to “de-mystify” the roles of microbial populations. Using the HUMAnN package [35], taxonomic abundances for R1 and R6 were used to infer metabolic processes for the two communities. Predicted features characterising either reactor were then identified using LEfSe [36], with complete metabolic HUMAnN and LDA outputs provided as supplementary data in S3 Table.

Diverse carbohydrate metabolism is likely to characterise R1, with the highest LDA effect scores (4.1–3.9, α = 0.006) for central carbohydrate metabolism and saccharide transport. Although carbohydrates are fundamental to all metabolism, the variety of metabolic pathways represented in these categories suggests that the R1 community utilises a more opportunistic and varied range of carbon sources, with significantly elevated predictions for the Entner-Doudoroff Pathway, Pentose Phosphate Pathway and Citrate Cycle (LDA effects: 3.18–3.42, α<0.05). Predicted markers for R1 also include transport of putrescine and spermidine, key components [90] in the formation and regulation of biofilms (LDA effect: 3.47–3.71, α = 0.006–0.011); and Type VI secretion systems which are likely to be used in competition for resources (LDA effect: 3.7, α = 0.034).

Metabolism of methane is a strong recurring prediction for R6 (LDA effect: 3.53–3.98, p = 0.006) with the emphasis on methanogenesis via methanol and acetate (LDA effect: 3.64 and 3.58 respectively, α = 0.006). However, the strongest predicted characteristics of R6 metabolism are transport of cobalt (LDA effect: 4, α = 0.006) and nickel (LDA effect: 4.2, α = 0.006). Cobalt is required for methylotrophic methanogenesis [57], while nickel is central to the final step of all methanogenic pathways [91,92]. There is good evidence in the literature indicating that methane production increases substantially when nickel and cobalt are added [93–95]. Increased archaeal ribosome metabolism (LDA effect: 3.64, α = 0.006) and reduction of quinones in energy metabolism (LDA effect: 3.52, α<0.02) are also predicted to differentiate metabolism in R6 from R1.

#### Constrained Correlation Analysis

Constrained Correlation Analysis (CCA) measured the relationships between community structure and time-points, and metabolism and time-points, in the context of specified ('constraining') process variables. Several process variables were inter-correlated, describing the same source of variation in the dataset. In particular, levels of TAN, alkalinity and total dissolve solids (TDS) were strongly inter-correlated (R = 0.80–0.95), as were B_eff_, biogas output and specific methane yield (SMY) (R = 0.81–0.97); and chloride, total salinity, chemical oxygen demand (COD), volatile solids (VS) and duration of trial (R = 0.81–0.97). As such, three governing aspects described the reactor communities: inhibitor accumulation, biogas activity, and trial duration.

#### CCA of community abundances

**Fig 3:**
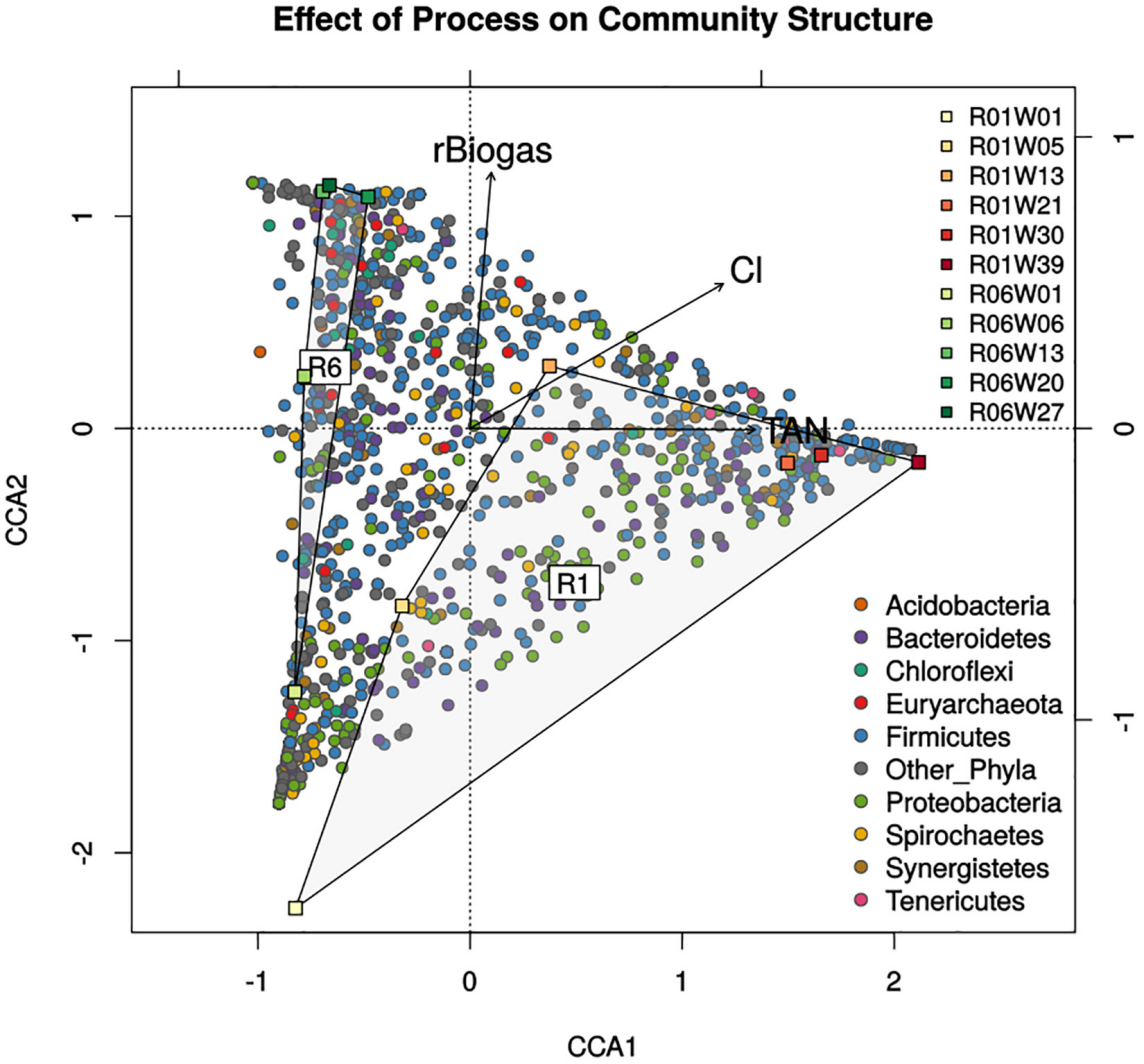
Levels of ammonia (TAN) and biogas best differentiate microbial communities between the two reactors. Microbial community structures diverged over time despite initial similarities (lower left quadrant), with R1 communities showing a stronger correlation with levels of ammonia across the X axis and R6 communities showing a stronger correlation with increasing biogas along the Y axis. The perpendicular relationship between biogas and ammonia (total ammoniacal nitrogen, ‘TAN’) suggests the two parameters act on community structure independently. Chloride (‘Cl’) levels show a weaker interaction with community structure, likely reflecting the accumulation of material and maturation of the reactor as the trial progresses.

CCA showed that levels of ammonia (specifically total ammoniacal nitrogen, TAN), chloride and raw biogas output had the strongest correlations with community make-up, with the most significant and non-redundant effects on taxonomic abundances (R = 0.50, significant after 999 permutations, VIF< 8). Together, these 3 parameters described 49.8% of variation in community abundance and allowed the major interactions defining these communities to be visualised via bi-plot (Fig 3) showing clear segregation between the two reactors. Although initial community and process similarities cause both Week1 samples to cluster, R1 and R6 time-points diverged along X and Y axes respectively, with clustering of later time-points showing established communities. Despite low OLR in R1, accumulation of TAN exceeded 5,000 mg/L in later time-points, and was the most strongly correlated inhibitor of biogas process (X axis). R6 time-points show negligible interaction with ammonia levels or overloading along the X axis, indicating the R6 community was not inhibited by TAN levels up to 3,000 mg/L. Instead, R6 correlates strongly with increasing biogas output, seen as distribution along the Y axis. Note that Week 13 of R1 correlated with biogas production (movement on Y axis) before R1 reached higher ammonia levels. Rising chloride concentrations correlate with both reactor setups, relating trial duration and a gradual accumulation of dissolved content. A stronger association with R1 is explained through a higher *U*. *lactuca* loading, with no clear inhibitory effects.

Correlations with OLR, reactor alkalinity (Alk) and total ammoniacal nitrogen (TAN) were up to 1.5 times stronger for *Archaea*, while pH, salinity, COD, VS% and Cl correlated to *Bacteria* more strongly (1.5–2 times). Curiously, the bacterial community was more than twice as correlated to B_eff_ as the archaeal community (R: 0.21 v 0.12), reflecting the specialised bacterial community involved in methanogenesis and relatively consistent methanogen components. A negative correlation between biogas output and biodiversity indices (R>-0.6) could potentially be explained through 'niche exclusion', where taxa unsuited to anaerobic digestion are out-competed by “better-equipped” taxa, causing a decrease in diversity. Excluded taxa are known to persist at low abundances and form important reservoirs of metabolic capability, invoked during shifts in reactor conditions [96–98].

#### CCA of predicted metabolic activities

**Fig 4:**
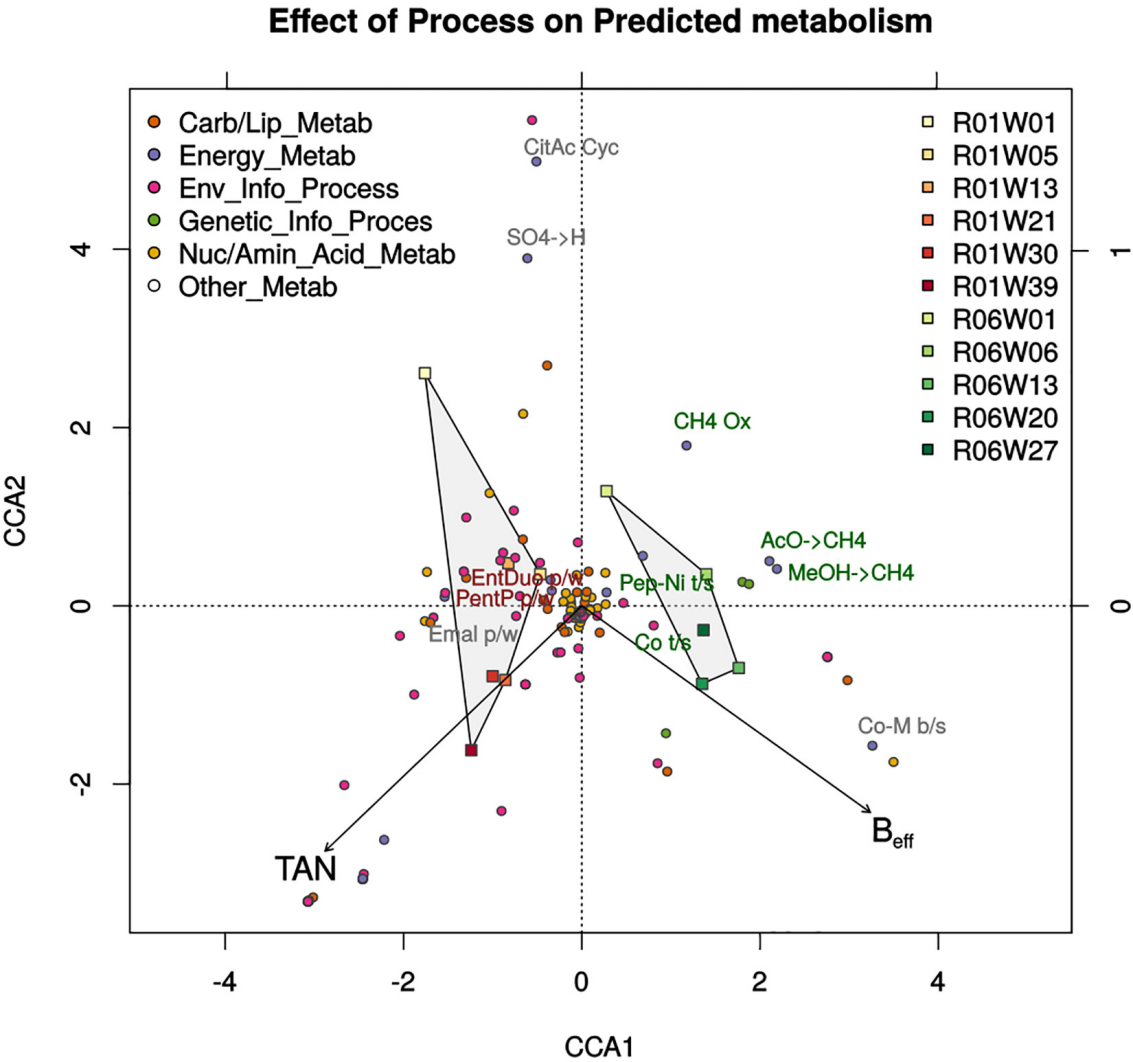
Ammonia levels (TAN) and biomethane conversion efficiency (B_eff_) best differentiate predicted metabolisms between R1 and R6. Carbon metabolisms segregate along the X axis, reflecting divergent environments under the contrasting reactor setups. R1 samples ordinate more closely with diverse carbon metabolism (Entner-Doudoroff: *EntDu p/w*; Pentose-Phosphate: *PentP p/w*; ethymalonyl: *Emal p/w*), while R6 samples ordinate strongly with methanogenic activities (Methanogenesis: *AcO*, *MeOH → CH4*; Co-Enzyme M biosynthesis: *Co-M b/s)*) and the uptake of trace elements (Cobalt: Co t/s; Nickel: *Pep-Ni t/s*). More diverse activities (Citric Acid Cycle: CitAc Cyc; sulphate reduction: *SO4->H;* methane oxidation: *CH4 Ox*) ordinate closer to earlier samples, suggesting metabolic activities detrimental to biogas production were excluded as reactor communities developed. Activities in green represent strongest predicted biomarkers for R6, activities in red represent strongest predicted biomarkers for R1.

CCA using predicted metabolic abundances showed strongest non-redundant correlations with TAN and B_eff_ (R = 0.50, VIF = 1, significant after 999 permutations). Ordination under these constraints (Fig 4) showed differences in energy metabolism along the X axis, with methanogenesis predictions related to R6 segregating from predicted alternative anaerobic metabolic pathways (Entner-Doudoroff, ethylmalonyl, and pentose-phosphate pathways) and carbon uptake pathways (multi-saccharide transport system) related to R1. Samples differentiated along the Y axis as reactors matured, with earlier metabolic diversity (e.g. sulphate reduction and transport, methane oxidation) absent in later samples as overall diversity decreased. Methanogenesis (acetate and methanol metabolism) and archaeal translation and transcription clearly associated with R6, while negatively correlating with TAN levels. Predictions for nickel and cobalt transport also associate with R6 time-points.

## Conclusion

Anaerobic digestion of *U*. *lactuca* appears to indirectly inhibit acetogenic and methanogenic processes, with ammonia showing the strongest causative correlation. At high *U*. *lactuca* volumes, decreasing OLR was not sufficient to recover the acetoclastic methanogens required to remove acetic acid and prevent overloading, nor to retain hydrogenotrophic methanogens. At lower *U*. *lactuca* volumes, the inhibition of acetogenesis caused *Methanosarcina* populations yields to shrink, affecting overall biogas yield. *U*. *lactuca* loading significantly affected community composition within reactors, with higher volumes characterised by diverse, facultatively anaerobic, and marine and halotolerant taxa, a lack of methanogens, and a predicted reliance on alternative carbon metabolism.

## Supporting Information

S1 Fig Rarefaction curves for pyrosequencing of R1 and R6 Communities. Rarefaction curves for rate of species observation (a), and the Shannon (b) and Chao1 (c) Diversity indices. Plateau'd curves indicate thorough and representative sampling of time-point communities. The Shannon Index is sensitive to the major community members, the Chao1 Index is more sensitive to diversity of rare species. Rarefaction curves indicate the major community members are well-characterised, but a large reservoir of low-abundance taxa remains undocumented.
(TIFF)

S2 Fig Relationships between communities displayed via UNIFRAC distances. UNIFRAC distances are a measure of similarity between communities, with more similar communities possessing a lower score, being more 'closely' related. Early communities (e.g. R01W01 & R06W01) were relatively similar, acclimatising to their respective feedstocks over time.
(JPG)

S1 Table Relative Taxonomic abundances for R1 and R6. Taxa are differentiated to the genus level; with abundances summed for lower taxonomic ranks.
(XLSX)

S2 Table Taxonomic Linear Discriminant Analysis (LDA) markers (via LEfSe [36]) for Ulva Digestion. Taxonomic abundances are related to either R1 or R6: the effectiveness of each taxon as a marker for either state is determined via its LDA effect size, as calculated in LEfSe [36], along with the significance of that effect (alpha value). A conservative LDA effect cut-off point of 3 used.
(XLSX)

S3 Table Predicted Metabolic Relative Abundances (via HUMAnN Package), and statistically significant Linear Discriminant Analysis (LDA) markers (via LEfSe). Metabolic abundances are inferred from taxonomic relative abundances via KEGG annotations [35], providing expected metabolic activities. The effectiveness of each metabolism as a marker is determined via its LDA effect size, as calculated in LEfSe [36], along with the significance of that effect (alpha value). A conservative LDA effect cut-off point of 3 used.
(XLSX)

## References

[1] Dave A, Huang Y, Rezvani S, McIlveen-Wright D, Novaes M, Hewitt N. Techno-economic assessment of biofuel development by anaerobic digestion of European marine cold-water seaweeds. Bioresour Technol. 2013 5;135:120–7. 10.1016/j.biortech.2013.01.005 23462594

[2] Vanegas CH, Bartlett J. Green energy from marine algae: biogas production and composition from the anaerobic digestion of Irish seaweed species. Environ Technol. 2013 8 1;34(15):2277–83. 24350482 10.1080/09593330.2013.765922

[3] Rodriguez C, Alaswad A, Mooney J, Prescott T, Olabi AG. Pre-treatment techniques used for anaerobic digestion of algae. Fuel Process Technol [Internet]. Epub 2015 Jun 29 [cited 2015 Oct 6]; Available from: http://www.sciencedirect.com/science/article/pii/S0378382015300527

[4] Allen E, Browne J, Hynes S, Murphy JD. The potential of algae blooms to produce renewable gaseous fuel. Waste Manag. 2013 11;33(11):2425–33. 10.1016/j.wasman.2013.06.017 23850117

[5] Briand X, Morand P. Anaerobic digestion of Ulva sp. 1. Relationship between Ulva composition and methanisation. J Appl Phycol. 1997 12;9(6):511–24.

[6] Morand P, Merceron M. Macroalgal Population and Sustainability. J Coast Res. 2005 9 1;1009–20.

[7] Percival E. The polysaccharides of green, red and brown seaweeds: Their basic structure, biosynthesis and function. Br Phycol J. 1979 6;14(2):103–17.

[8] Wong KH, Cheung PC. Nutritional evaluation of some subtropical red and green seaweeds: Part I—proximate composition, amino acid profiles and some physico-chemical properties. Food Chem. 2000;71(4):475–82.

[9] Koch C, Müller S, Harms H, Harnisch F. Microbiomes in bioenergy production: From analysis to management. Curr Opin Biotechnol. 2014 6;27:65–72. 10.1016/j.copbio.2013.11.006 24863898

[10] Carballa M, Regueiro L, Lema JM. Microbial management of anaerobic digestion: exploiting the microbiome-functionality nexus. Curr Opin Biotechnol. 2015 6;33:103–11. 10.1016/j.copbio.2015.01.008 25682574

[11] Solli L, Håvelsrud OE, Horn SJ, Rike AG. A metagenomic study of the microbial communities in four parallel biogas reactors. Biotechnol Biofuels. 2014 10 14;7(1):146 10.1186/s13068-014-0146-2 25328537 PMC4200192

[12] St-Pierre B, Wright A-DG. Comparative metagenomic analysis of bacterial populations in three full-scale mesophilic anaerobic manure digesters. Appl Microbiol Biotechnol. 2014 3;98(6):2709–17. 10.1007/s00253-013-5220-3 24085391

[13] Sundberg C, Soud WA Al-, Larsson M, Alm E, Yekta SS, Svensson BH, et al 454 pyrosequencing analyses of bacterial and archaeal richness in 21 full-scale biogas digesters. FEMS Microbiol Ecol. 2013 9;85(3):612–26. 10.1111/1574-6941.12148 23678985

[14] Wilkins D, Lu X-Y, Shen Z, Chen J, Lee PKH. Pyrosequencing of mcrA and Archaeal 16S rRNA Genes Reveals Diversity and Substrate Preferences of Methanogen Communities in Anaerobic Digesters. Appl Environ Microbiol. 2015 1 15;81(2):604–13. 10.1128/AEM.02566-14 25381241 PMC4277577

[15] Pope PB, Vivekanand V, Eijsink VGH, Horn SJ. Microbial community structure in a biogas digester utilizing the marine energy crop Saccharina latissima. 3 Biotech. 2013 10;3(5):407–14. 10.1007/s13205-012-0097-x PMC3781269 28324331

[16] Wirth R, Lakatos G, Maróti G, Bagi Z, Minárovics J, Nagy K, et al Exploitation of algal-bacterial associations in a two-stage biohydrogen and biogas generation process. Biotechnol Biofuels. 2015;8(1):59. 25873997 10.1186/s13068-015-0243-x PMC4395902

[17] Hinks J, Edwards S, Sallis PJ, Caldwell GS. The steady state anaerobic digestion of Laminaria hyperborea–Effect of hydraulic residence on biogas production and bacterial community composition. Bioresour Technol. 2013 9;143:221–30. 10.1016/j.biortech.2013.05.124 23792760

[18] Allen E, Wall DM, Herrmann C, Murphy JD. Investigation of the optimal percentage of green seaweed that may be co-digested with dairy slurry to produce gaseous biofuel. Bioresour Technol. 2014 10;170:436–44. 10.1016/j.biortech.2014.08.005 25164335

[19] Nordmann W. Die Überwachung der Schlammfaulung. KA-Informationen für das Betriebspersonal, Beilage zur Korrespondenz Abwasser. 1977;3/77. German.

[20] Baker GC, Smith JJ, Cowan DA. Review and re-analysis of domain-specific 16S primers. J Microbiol Methods. 2003 12;55(3):541–55. 14607398 10.1016/j.mimet.2003.08.009

[21] Bragg L, Stone G, Imelfort M, Hugenholtz P, Tyson GW. Fast, accurate error-correction of amplicon pyrosequences using Acacia. Nat Meth. 2012 5;9(5):425–6. 10.1038/nmeth.1990 22543370

[22] Caporaso JG, Kuczynski J, Stombaugh J, Bittinger K, Bushman FD, Costello EK, et al QIIME allows analysis of high-throughput community sequencing data. Nat Methods. 2010;7(5):335–6. 10.1038/nmeth.f.303 20383131 PMC3156573

[23] Edgar RC. Search and clustering orders of magnitude faster than BLAST. Bioinformatics. 2010 10 1;26(19):2460–1. 10.1093/bioinformatics/btq461 20709691

[24] Quast C, Pruesse E, Yilmaz P, Gerken J, Schweer T, Yarza P, et al The SILVA ribosomal RNA gene database project: improved data processing and web-based tools. Nucleic Acids Res. 2013 1 1;41(D1):D590–6. 23193283 10.1093/nar/gks1219 PMC3531112

[25] Caporaso JG, Bittinger K, Bushman FD, DeSantis TZ, Andersen GL, Knight R. PyNAST: a flexible tool for aligning sequences to a template alignment. Bioinformatics. 2010;26(2):266–7. 10.1093/bioinformatics/btp636 19914921 PMC2804299

[26] Wang Q, Garrity GM, Tiedje JM, Cole JR. Naive Bayesian classifier for rapid assignment of rRNA sequences into the new bacterial taxonomy. Appl Environ Microbiol. 2007;73(16):5261–7. 17586664 10.1128/AEM.00062-07 PMC1950982

[27] Price MN, Dehal PS, Arkin AP. FastTree 2–approximately maximum-likelihood trees for large alignments. PloS One. 2010;5(3):e9490 10.1371/journal.pone.0009490 20224823 PMC2835736

[28] Lozupone C, Knight R. UniFrac: a new phylogenetic method for comparing microbial communities. Appl Environ Microbiol. 2005;71(12):8228–35. 16332807 10.1128/AEM.71.12.8228-8235.2005 PMC1317376

[29] Vázquez-Baeza Y, Pirrung M, Gonzalez A, Knight R. EMPeror: a tool for visualizing high-throughput microbial community data. Structure. 2013;585:20. 10.1186/2047-217X-2-16 PMC4076506 24280061

[30] R Core Team. R: A Language and Environment for Statistical Computing [Internet]. Vienna, Austria: R Foundation for Statistical Computing; 2013 Available from: http://www.R-project.org/

[31] Oksanen J, Blanchet FG, Kindt R, Legendre P, Minchin PR, O’Hara RB, et al. vegan: Community Ecology Package [Internet]. 2014. Available from: http://R-Forge.R-project.org/projects/vegan/

[32] McMurdie PJ, Holmes S. phyloseq: An R Package for Reproducible Interactive Analysis and Graphics of Microbiome Census Data. Watson M, editor. PLoS ONE. 2013 4 22;8(4):e61217 10.1371/journal.pone.0061217 23630581 PMC3632530

[33] DeSantis TZ, Hugenholtz P, Larsen N, Rojas M, Brodie EL, Keller K, et al Greengenes, a chimera-checked 16S rRNA gene database and workbench compatible with ARB. Appl Environ Microbiol. 2006;72(7):5069–72. 16820507 10.1128/AEM.03006-05 PMC1489311

[34] Kanehisa M, Goto S, Sato Y, Kawashima M, Furumichi M, Tanabe M. Data, information, knowledge and principle: back to metabolism in KEGG. Nucleic Acids Res. 2014 1 1;42(D1):D199–205. 24214961 10.1093/nar/gkt1076 PMC3965122

[35] Abubucker S, Segata N, Goll J, Schubert AM, Izard J, Cantarel BL, et al Metabolic Reconstruction for Metagenomic Data and Its Application to the Human Microbiome. PLoS Comput Biol. 2012 6 13;8(6):e1002358 10.1371/journal.pcbi.1002358 22719234 PMC3374609

[36] Segata N, Izard J, Waldron L, Gevers D, Miropolsky L, Garrett WS, et al Metagenomic biomarker discovery and explanation. Genome Biol. 2011;12(6):R60 10.1186/gb-2011-12-6-r60 21702898 PMC3218848

[37] Giardine B, Riemer C, Hardison RC, Burhans R, Elnitski L, Shah P, et al Galaxy: A platform for interactive large-scale genome analysis. Genome Res. 2005 10 1;15(10):1451–5. 16169926 10.1101/gr.4086505 PMC1240089

[38] Blankenberg D, Kuster GV, Coraor N, Ananda G, Lazarus R, Mangan M, et al Galaxy: A Web-Based Genome Analysis Tool for Experimentalists In: Current Protocols in Molecular Biology [Internet]. John Wiley & Sons, Inc.; 2001 [cited 2015 Aug 6]. Available from: http://onlinelibrary.wiley.com/doi/10.1002/0471142727.mb1910s89/abstract 10.1002/0471142727.mb1910s89 PMC4264107 20069535

[39] Goecks J, Nekrutenko A, Taylor J. Galaxy: a comprehensive approach for supporting accessible, reproducible, and transparent computational research in the life sciences. Genome Biol. 2010;11(8):R86 10.1186/gb-2010-11-8-r86 20738864 PMC2945788

[40] Santana RH, Catão ECP, Lopes FAC, Constantino R, Barreto CC, Krüger RH. The Gut Microbiota of Workers of the Litter-Feeding Termite *Syntermes wheeleri* (*Termitidae*: *Syntermitinae*): Archaeal, Bacterial, and Fungal Communities. Microb Ecol. 2015 8;70(2):545–56. 10.1007/s00248-015-0581-z 25749937

[41] Zhang J, Zhang Y, Quan X, Chen S. Enhancement of anaerobic acidogenesis by integrating an electrochemical system into an acidogenic reactor: Effect of hydraulic retention times (HRT) and role of bacteria and acidophilic methanogenic Archaea. Bioresour Technol. 2015 3;179:43–9. 10.1016/j.biortech.2014.11.102 25514401

[42] Mhuantong W, Wongwilaiwalin S, Laothanachareon T, Eurwilaichitr L, Tangphatsornruang S, Boonchayaanant B, et al Survey of Microbial Diversity in Flood Areas during Thailand 2011 Flood Crisis Using High-Throughput Tagged Amplicon Pyrosequencing. PLoS ONE. 2015 5 28;10(5):e0128043 10.1371/journal.pone.0128043 26020967 PMC4447364

[43] Green SJ, Venkatramanan R, Naqib A. Deconstructing the Polymerase Chain Reaction: Understanding and Correcting Bias Associated with Primer Degeneracies and Primer-Template Mismatches. PLoS ONE [Internet]. 2015 5 21 [cited 2015 Sep 24];10(5). 10.1371/journal.pone.0128122 PMC4440812 25996930

[44] McCarty PL, McKinney RE. Volatile acid toxicity in anaerobic digestion. J Water Pollut Control Fed. 1961;223–32.

[45] Wijekoon KC, Visvanathan C, Abeynayaka A. Effect of organic loading rate on VFA production, organic matter removal and microbial activity of a two-stage thermophilic anaerobic membrane bioreactor. Bioresour Technol. 2011 5;102(9):5353–60. 10.1016/j.biortech.2010.12.081 21256737

[46] Williams J, Williams H, Dinsdale R, Guwy A, Esteves S. Monitoring methanogenic population dynamics in a full-scale anaerobic digester to facilitate operational management. Bioresour Technol. 2013 7;140:234–42. 10.1016/j.biortech.2013.04.089 23707910

[47] Griffin ME, McMahon KD, Mackie RI, Raskin L. Methanogenic population dynamics during start-up of anaerobic digesters treating municipal solid waste and biosolids. Biotechnol Bioeng. 1998;57(3):342–55. 10099211 10.1002/(sici)1097-0290(19980205)57:3<342::aid-bit11>3.0.co;2-i

[48] Pullammanappallil PC, Chynoweth DP, Lyberatos G, Svoronos SA. Stable performance of anaerobic digestion in the presence of a high concentration of propionic acid. Bioresour Technol. 2001;78(2):165–9. 11333036 10.1016/s0960-8524(00)00187-5

[49] Seppälä M, Pyykkönen V, Väisänen A, Rintala J. Biomethane production from maize and liquid cow manure–Effect of share of maize, post-methanation potential and digestate characteristics. Fuel. 2013 5;107(0):209–16.

[50] Sprott GD, Shaw KM, Jarrell KF. Ammonia/potassium exchange in methanogenic bacteria. J Biol Chem. 1984;259(20):12602–8. 6490632

[51] Fotidis IA, Karakashev D, Kotsopoulos TA, Martzopoulos GG, Angelidaki I. Effect of ammonium and acetate on methanogenic pathway and methanogenic community composition. FEMS Microbiol Ecol. 2013 1;83(1):38–48. 10.1111/j.1574-6941.2012.01456.x 22809020

[52] Calli B, Mertoglu B, Inanc B, Yenigun O. Methanogenic diversity in anaerobic bioreactors under extremely high ammonia levels. Enzyme Microb Technol. 2005 9;37(4):448–55.

[53] Sprott GD. Ammonia toxicity in pure cultures of methanogenic bacteria. Syst Appl Microbiol. 1986 5;Volume 7(Issues 2–3):Pages 358–63.

[54] Chen Y, Cheng JJ, Creamer KS. Inhibition of anaerobic digestion process: A review. Bioresour Technol. 2008 7;99(10):4044–64. 17399981 10.1016/j.biortech.2007.01.057

[55] Yenigün O, Demirel B. Ammonia inhibition in anaerobic digestion: A review. Process Biochem. 2013 5;48(5–6):901–11.

[56] McCarty PL, McKinney RE. Salt toxicity in anaerobic digestion. J Water Pollut Control Fed. 1961;399–415.

[57] Gottschalk G, Thauer RK. The Na+-translocating methyltransferase complex from methanogenic archaea. Biochim Biophys Acta BBA—Bioenerg. 2001 5 1;1505(1):28–36. 10.1016/s0005-2728(00)00274-7 11248186

[58] Feijoo G, Soto M, Méndez R, Lema JM. Sodium inhibition in the anaerobic digestion process: Antagonism and adaptation phenomena. Enzyme Microb Technol. 1995 2;(17):180–8.

[59] Rinzema A, Boone M, van Knippenberg K, Lettinga G. Bactericidal Effect of Long Chain Fatty Acids in Anaerobic Digestion. Water Environ Res. 1994 1 1;66(1):40–9.

[60] Conklin A, Stensel HD, Ferguson J. Growth Kinetics and Competition between Methanosarcina and Methanosaeta in Mesophilic Anaerobic Digestion. Water Environ Res. 2006 5 1;78(5):486–96. 16752610 10.2175/106143006x95393

[61] Hori T, Haruta S, Ueno Y, Ishii M, Igarashi Y. Dynamic Transition of a Methanogenic Population in Response to the Concentration of Volatile Fatty Acids in a Thermophilic Anaerobic Digester. Appl Environ Microbiol. 2006 2 1;72(2):1623–30. 16461718 10.1128/AEM.72.2.1623-1630.2006 PMC1392901

[62] De Vrieze J, Hennebel T, Boon N, Verstraete W. Methanosarcina: The rediscovered methanogen for heavy duty biomethanation. Bioresour Technol. 2012 5;112:1–9. 10.1016/j.biortech.2012.02.079 22418081

[63] Huser BA, Wuhrmann K, Zehnder AJB. Methanothrix soehngenii gen. nov. sp. nov., a New Acetotrophic Non-hydrogen-oxidizing Methane Bacterium. Arch Microbiol. 1982 7;132(1):1–9. 10.1007/BF00407022 6769415

[64] Oren A. The Family Methanosarcinaceae In: The Prokaryotes—Other Major Lineages of Bacteria and the Archaea. 4th ed. Springer-Verlag Berlin Heidelberg; 2014 p. 1028.

[65] Romesser JA, Wolfe RS, Mayer F, Spiess E, Walther-Mauruschat A. Methanogenium, a new genus of marine methanogenic bacteria, and characterization ofMethanogenium cariaci sp. nov. andMethanogenium marisnigri sp. nov. Arch Microbiol. 1979 5;121(2):147–53.

[66] Dridi B, Fardeau M-L, Ollivier B, Raoult D, Drancourt M. Methanomassiliicoccus luminyensis gen. nov., sp. nov., a methanogenic archaeon isolated from human faeces. Int J Syst Evol Microbiol. 2012 8 1;62(Pt 8):1902–7. 10.1099/ijs.0.033712-0 22859731

[67] Whitford MF, Teather RM, Forster RJ. Phylogenetic analysis of methanogens from the bovine rumen. BMC Microbiol. 2001;1(1):5. 11384509 10.1186/1471-2180-1-5 PMC32158

[68] Ohkuma M, Noda S, Kudo T. Phylogenetic relationships of symbiotic methanogens in diverse termites. FEMS Microbiol Lett. 1999;171(2):147–53. 10077839 10.1111/j.1574-6968.1999.tb13425.x

[69] Schlüter A, Bekel T, Diaz NN, Dondrup M, Eichenlaub R, Gartemann K-H, et al The metagenome of a biogas-producing microbial community of a production-scale biogas plant fermenter analysed by the 454-pyrosequencing technology. J Biotechnol. 2008 8;136(1–2):77–90. 10.1016/j.jbiotec.2008.05.008 18597880

[70] Kröber M, Bekel T, Diaz NN, Goesmann A, Jaenicke S, Krause L, et al Phylogenetic characterization of a biogas plant microbial community integrating clone library 16S-rDNA sequences and metagenome sequence data obtained by 454-pyrosequencing. J Biotechnol. 2009 6;142(1):38–49. 10.1016/j.jbiotec.2009.02.010 19480946

[71] Claesson MJ, O’Sullivan O, Wang Q, Nikkilä J, Marchesi JR, Smidt H, et al Comparative Analysis of Pyrosequencing and a Phylogenetic Microarray for Exploring Microbial Community Structures in the Human Distal Intestine. Ahmed N, editor. PLoS ONE. 2009 8 20;4(8):e6669 10.1371/journal.pone.0006669 19693277 PMC2725325

[72] Nyonyo T, Shinkai T, Mitsumori M. Improved culturability of cellulolytic rumen bacteria and phylogenetic diversity of culturable cellulolytic and xylanolytic bacteria newly isolated from the bovine rumen. FEMS Microbiol Ecol. 2014 6;88(3):528–37. 10.1111/1574-6941.12318 24612331

[73] Grabowski A. Petrimonas sulfuriphila gen. nov., sp. nov., a mesophilic fermentative bacterium isolated from a biodegraded oil reservoir. Int J Syst Evol Microbiol. 2005 5 1;55(3):1113–21. 15879242 10.1099/ijs.0.63426-0

[74] Chen S, Dong X. Proteiniphilum acetatigenes gen. nov., sp. nov., from a UASB reactor treating brewery wastewater. Int J Syst Evol Microbiol. 2005 11 1;55(6):2257–61. 16280479 10.1099/ijs.0.63807-0

[75] Li A, Chu Y, Wang X, Ren L, Yu J, Liu X, et al A pyrosequencing-based metagenomic study of methane-producing microbial community in solid-state biogas reactor. Biotechnol Biofuels [Internet]. 2013 [cited 2015 May 29];6(3). 10.1186/1754-6834-6-3 PMC3618299 23320936

[76] Delbes C, Moletta R, Godon J-J. Monitoring of activity dynamics of an anaerobic digester bacterial community using 16S rRNA polymerase chain reaction–single-strand conformation polymorphism analysis. Environ Microbiol. 2000;2(5):506–15. 11233159 10.1046/j.1462-2920.2000.00132.x

[77] Fernández A, Huang S, Seston S, Xing J, Hickey R, Criddle C, et al How Stable Is Stable? Function versus Community Composition. Appl Environ Microbiol. 1999 8 1;65(8):3697–704. 10427068 10.1128/aem.65.8.3697-3704.1999 PMC91553

[78] Lee S-H, Park J-H, Kang H-J, Lee YH, Lee TJ, Park H-D. Distribution and abundance of Spirochaetes in full-scale anaerobic digesters. Bioresour Technol. 2013 10;145:25–32. 10.1016/j.biortech.2013.02.070 23562175

[79] Jumas-Bilak E, Marchandin H. The Phylum Synergistetes In: The Prokarytoes—Other Major Lineages of Bacterialand the Archaea. 4th ed. Springer-Verlag Berlin Heidelberg; 2014.

[80] Baena S, Fardeau ML, Labat M, Ollivier B, Thomas P, Garcia JL, et al Aminobacterium colombiensegen. nov. sp. nov., an amino acid-degrading anaerobe isolated from anaerobic sludge. Anaerobe. 1998 10;4(5):241–50. 16887649 10.1006/anae.1998.0170

[81] Baena S, Fardeau M-L, Labat M, Ollivier B, Garcia J-L, Patel B. Aminobacterium mobile sp. nov., a new anaerobic amino-acid-degrading bacterium. Int J Syst Evol Microbiol. 2000;50(1):259–64. 10826812 10.1099/00207713-50-1-259

[82] Yamada T. Anaerolinea thermolimosa sp. nov., Levilinea saccharolytica gen. nov., sp. nov. and Leptolinea tardivitalis gen. nov., sp. nov., novel filamentous anaerobes, and description of the new classes Anaerolineae classis nov. and Caldilineae classis nov. in the bacterial phylum Chloroflexi. Int J Syst Evol Microbiol. 2006 6 1;56(6):1331–40. 16738111 10.1099/ijs.0.64169-0

[83] Martini M, Marcone C, Lee I-M, Firrao G. The Family Acholeplasmataceae (including Phytoplasmas) In: The Prokarytoes—Firmicutes and Tenericutes. 4th ed. Springer-Verlag Berlin Heidelberg; 2014.

[84] Tauch A, Sandbote J. The Family Corynebacteriaceae In: The Prokaryotes—Actinobacteria. 4th ed. Springer-Verlag Berlin Heidelberg; 2014.

[85] Lee KCY, Dunfield PF, Stott MB. The Phylum Armatimonadetes In: The Prokaryotes—Other Major Lineages of Bacteria and the Archaea. 4th ed. Springer-Verlag Berlin Heidelberg; 2014.

[86] Teixiera LM, Merquior VLC. The Family Moraxellaceae In: The Prokarytoes—Gammaproteobacteria. 4th ed. Springer-Verlag Berlin Heidelberg; 2014.

[87] Chumpitazi BP, Cope JL, Hollister EB, Tsai CM, McMeans AR, Luna RA, et al Randomised clinical trial: gut microbiome biomarkers are associated with clinical response to a low FODMAP diet in children with the irritable bowel syndrome. Aliment Pharmacol Ther. 2015 8 1;42(4):418–27. 10.1111/apt.13286 26104013 PMC4514898

[88] Loudon AH, Woodhams DC, Parfrey LW, Archer H, Knight R, McKenzie V, et al Microbial community dynamics and effect of environmental microbial reservoirs on red-backed salamanders (Plethodon cinereus). ISME J. 2014 4;8(4):830–40. 10.1038/ismej.2013.200 24335825 PMC3960541

[89] Jiménez DJ, Dini-Andreote F, van Elsas JD. Metataxonomic profiling and prediction of functional behaviour of wheat straw degrading microbial consortia. Biotechnol Biofuels. 2014 6 12;7:92 10.1186/1754-6834-7-92 24955113 PMC4064818

[90] Karatan E, Michael AJ. A wider role for polyamines in biofilm formation. Biotechnol Lett. 2013 11;35(11):1715–7. 10.1007/s10529-013-1286-3 23881324

[91] Pelmenschikov V, Blomberg MRA, Siegbahn PEM, Crabtree RH. A Mechanism from Quantum Chemical Studies for Methane Formation in Methanogenesis. J Am Chem Soc. 2002 4;124(15):4039–49. 11942842 10.1021/ja011664r

[92] Thauer RK, Kaster A-K, Goenrich M, Schick M, Hiromoto T, Shima S. Hydrogenases from Methanogenic Archaea, Nickel, a Novel Cofactor, and H _2_ Storage. Annu Rev Biochem. 2010 6 7;79(1):507–36. 20235826 10.1146/annurev.biochem.030508.152103

[93] Murray WD, van den Berg L. Effects of Nickel, Cobalt, and Molybdenum on Performance of Methanogenic Fixed-Film Reactor. Appl Environ Microbiol. 1981 9;42(3):502–5. 16345846 10.1128/aem.42.3.502-505.1981 PMC244044

[94] Gonzalez-Gil G, Kleerebezem R, Lettinga G. Effects of nickel and cobalt on kinetics of methanol conversion by methanogenic sludge as assessed by on-line CH4 monitoring. Appl Environ Microbiol. 1999;65(4):1789–93. 10103284 10.1128/aem.65.4.1789-1793.1999 PMC91254

[95] Zandvoort MH, van Hullebusch ED, Fermoso FG, Lens PNL. Trace Metals in Anaerobic Granular Sludge Reactors: Bioavailability and Dosing Strategies. Eng Life Sci. 2006 6;6(3):293–301.

[96] Werner JJ, Knights D, Garcia ML, Scalfone NB, Smith S, Yarasheski K, et al Bacterial community structures are unique and resilient in full-scale bioenergy systems. Proc Natl Acad Sci. 2011 3 8;108(10):4158–63. 10.1073/pnas.1015676108 21368115 PMC3053989

[97] Nelson MC, Morrison M, Schanbacher F, Yu Z. Shifts in microbial community structure of granular and liquid biomass in response to changes to infeed and digester design in anaerobic digesters receiving food-processing wastes. Bioresour Technol. 2012 3;107:135–43. 10.1016/j.biortech.2011.12.070 22257856

[98] Briones A, Raskin L. Diversity and dynamics of microbial communities in engineered environments and their implications for process stability. Curr Opin Biotechnol. 2003 6;14(3):270–6. 12849779 10.1016/s0958-1669(03)00065-x

