# Supplementary figures and images for: Methanosarcina Play an Important Role in Anaerobic Co-Digestion of the Seaweed Ulva lactuca: Taxonomy and Predicted Metabolism of Functional Microbial Communities
Source: PLoS One. 2015 Nov 10;10(11):e0142603. doi: 10.1371/journal.pone.0142603 (PMC4640829; doi:10.1371/journal.pone.0142603)

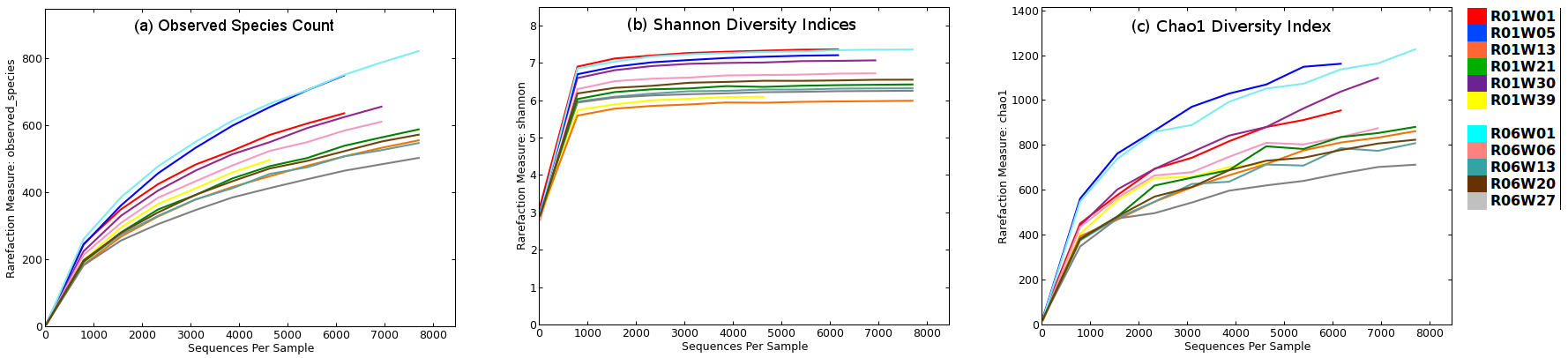

Supplement: S1 Fig — Rarefaction curves for rate of species observation (a), and the Shannon (b) and Chao1 (c) Diversity indices. Plateau'd curves indicate thorough and representative sampling of time-point communities. The Shannon Index is sensitive to the major community members, the Chao1 Index is more sensitive to diversity of rare species. Rarefaction curves indicate the major community members are well-characterised, but a large reservoir of low-abundance taxa remains undocumented. (TIFF) [file pone.0142603.s001.tiff]

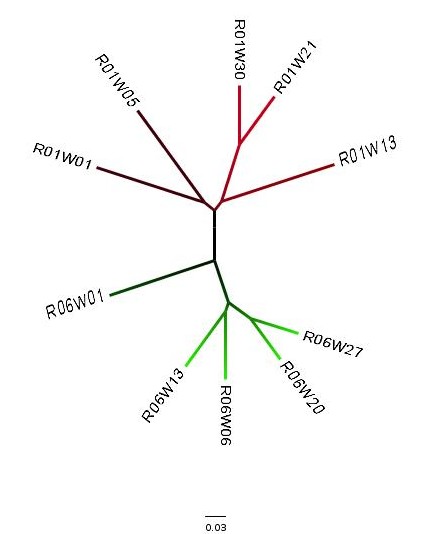

Supplement: S2 Fig — UNIFRAC distances are a measure of similarity between communities, with more similar communities possessing a lower score, being more 'closely' related. Early communities (e.g. R01W01 & R06W01) were relatively similar, acclimatising to their respective feedstocks over time. (JPG) [file pone.0142603.s002.jpg]
